# Inside the horn of plenty: Leaf-mining micromoth manipulates its host plant to obtain unending food provisioning

**DOI:** 10.1371/journal.pone.0209485

**Published:** 2018-12-21

**Authors:** Antoine Guiguet, Akihisa Hamatani, Taisuke Amano, Seiji Takeda, Carlos Lopez-Vaamonde, David Giron, Issei Ohshima

**Affiliations:** 1 Institut de Recherche sur la Biologie de l’Insecte, UMR 7261, CNRS/Université de Tours, UFR Sciences et Techniques, Tours, France; 2 Department of Life and Environmental Sciences, Kyoto Prefectural University, Kyoto, Japan; 3 Cell and Genome Biology, Graduate School of Life and Environmental Sciences, Kyoto Prefectural University, Kyoto, Japan; 4 Biotechnology Research Department, Kyoto Prefectural Agriculture Forestry and Fisheries Technology Center, Seika, Soraku–gun, Kyoto, Japan; 5 INRA, UR0633 Zoologie Forestière, Orléans, France; Chinese Academy of Agricultural Sciences Institute of Plant Protection, CHINA

## Abstract

Leaves represent the main resource for herbivorous insects and their performances are mainly a function of leaf nutritional quality. Two feeding strategies are known to optimize the exploitation of leaf resources: leaf-miners that selectively feed on tissues of high nutritional quality and gall-inducers that induce the development of a new tissue showing an enhanced nutritional value. Some leaf-miners are known to also manipulate their nutritional environment, but do not affect plant development. Cases of callus proliferation in leaf-mines have been reported, however, the direct role of the insect in the formation of additional plant cells and the nutritional function of this tissue have never been established. Using an experimental approach, we show that leaf-mining larvae of micromoth, *Borboryctis euryae* (Lepidoptera: Gracillariidae), that grow on *Eurya japonica* (Pentaphylacaceae), actively induce callus proliferation within their leaf-mine at the fourth instar. We experimentally demonstrated that, at this developmental stage, the larva feeds exclusively on this newly formed tissue and feeding of the tissue is essential for completing larval stage. Phenological census revealed considerable expansion and variation of fourth instar duration caused by the continuous production of callus. We propose here the “cornucopia” hypothesis which states that the newly produced callus induced by the leaf-mining larvae provides virtually unending nourishment, which in turn allows flexible larval development time. This represents the first example of a leaf-miner manipulating plant development to its benefit, like a gall-inducer. We propose to name this life style “mine-galler”.

## Introduction

Plant quality is one of the main drivers in phytophagous insect performance [1]. The quality of plants for herbivores is mostly defined by the nutritional content and the physical and chemical defenses. In turn, plant nutritional quality is influenced by several environmental factors such as soil fertility, light condition, temperature, water deficit and seasonality [2,3]. In addition, the low quality of host plants can be an adaptive trait selected upon the high selective pressure exerted by insects. Many plants contain secondary metabolites that represent a defensive strategy, making them unsuitable for most herbivores [4]. Moreover, sublethal effects of low nutritional quality or low digestibility of plant tissues are also considered as defenses against herbivory. In particular, the slow-growth high-mortality hypothesis predicts that prolonged larval development, due to a low quality of the host-plant, increases the mortality of herbivores from their natural enemies [5]. Although the general validity of this hypothesis is still debated [6,7], it could explain the strategies developed by some insect species to decrease the impact of whole plant quality on their developmental time. In particular, two feeding strategies are generally considered as adaptation to optimize the nutritional quality of the exploited plant tissues: gall-inducing and leaf-mining.

Leaf-mining and gall-inducing are two endophagous life-styles differing by the insect capacity to manipulate the physiology of the host plant: whereas leaf-miners consume plant tissue by dwelling inside it, gallers induce growth and differentiation of new plant tissues. In this newly formed structure, gall-inducing insects are often able to induce the formation of a highly differentiated tissue, called nutritive tissue, which shows both an enhanced nutritive quality and contains less chemical defenses [8]. On the contrary, leaf-miners are defined as feeding only on pre-existing tissues. One of the hypothesis about the adaptive value of this life-style predicts that miners select tissues with high nutrient content and low chemical and structural defense content [9]. This so-called selective-feeding hypothesis has been verified in some cases [10], but some leaf-miners remain sensitive to decrease in host plant quality suggesting limits to this strategy [11].

This binary view defining the gallers versus miners, however, is challenged by the ability of some leaf miners to manipulate their nutritional environment. For example, the green-island phenotype induced by *Phyllonorycter* species on apple tree leaves allows maintenance of plant tissue quality in a degenerating context [12]. Several insects are also able to induce a cell proliferation inside leaf-mines that may play a role in their nutrition, like for gallers [13]. However, contrary to galls, the role of the leaf-miner in the development of this undifferentiated tissue, called callus, within the mine and its nutritive significance for the insect are not well understood. It has been suggested that the callus growth is caused by the physical conditions inside the mine, in particular a high moisture level, rather than the result of a direct effect of the insect on the plant [14]. On the other hand, it has been shown that callus formation could be stimulated by larval frass deposition, but without testing the nutritive role of this newly formed tissue [15]. Some studies found that the larva feeds on the callus but with no evidence that the observed cell growth is directly induced by the insect [16]. Thus, to date, the insect role in callus formation in the mine is still unclear and the nutritive value of this newly formed tissue for the insect remains to be established.

Here we study the Japanese leaf-mining micromoth *Borboryctis euryae* Kumata and Kuroko (Lepidoptera, Gracillariidae), whose larvae feed on *Eurya japonica* (Pentaphylacaceae), also called “hisakaki”. As indicated by its genus name (“borbos” means “swollen”, and “oryctis” means “mine”), the gallery formed inside a leaf by its larva is inflated. First, we verified that the mine inflation was caused by a callus proliferation using a histological approach. The larval stage corresponding to the initiation of the mine inflation (callus) was identified, and the callus (mass) were also quantified. Then, we designed manipulative experiments to address the following questions: (i) Does the callus growth turn the mined-leaf into a sink organ, like gall development does? (ii) Does the insect feed on the callus, and if yes, is it a facultative or an obligatory feeding source (like a gall)? (iii) Does *B*. *euryae* larvae directly induce the callus development in the mine, or is it indirectly stimulated by the physical conditions inside the mine? Finally, the link between the feeding behavior of *B*. *euryae* larvae and its developmental phenology is discussed to assess the potential benefits of callus proliferation within the leaf mine.

## Material and methods

### Field sampling

All insect samples were collected from or observed in the wild forest in Kibogaoka Cultural Park, (35˚05’ N, 136˚08’ E; altitude 180–230 m) or Yukinoyama Historical Park (35˚07’ N, 136˚15’ E; altitude 110–130 m), Shiga prefecture, Japan. Both study areas are secondary forests of both deciduous and evergreen vegetation, dominated by konara (*Quercus serrata*, Fagaceae), soyogo (*Ilex pedunculosa*, Aquifoliaceae), chinese sumac (*Rhus javanica*, Anacardiaceae) and hisakaki (*Eurya japonica*, Pentaphylacaceae), the host plant of *B*. *euryae*. The climate of the study site is a temperate type having hot and humid summers and moderately cold and dry winters (see S1 Table and S1 Fig for details).

### Laboratory rearing

To assess the dynamics of the mine inflation, we established a lab rearing of *B*. *euryae*. Leaf-mines of late larval instars (fourth and fifth) were collected and reared them into adults in the laboratory as described in [17]. A single pair of male and female adults was introduced into a centrifuge tube (118-mm long, 28-mm diameter) containing a young leaf. After starting oviposition, each female was transferred to a mesh bag or a mesh cage wrapping twigs with young leaves of potted *E*. *japonica* in the greenhouse at Kyoto Prefectural University, Kyoto, Japan. Since females lay their eggs at night, oviposition was checked the next morning and recorded the development of mines every day after hatching. Changes of larval instars were recorded by counting the molting of the head-capsules of previous developmental stages.

### Histological study of the early steps of the mine inflation

To verify if the mine inflation is due to the proliferation of callus, histological cross-sections of mines collected on the field were made before and after appearance of inflation symptoms. Therefore, cross-sections were made at third instar and early fourth instar (one day after molting) when the mine is still flat, and at fourteen days after beginning of fourth instar, when the mine inflation is clearly visible. Cross-section of the mine of a fifth instars was made to compare the feeding of this instar mode with previous development stages (for description of the method, see section below “*General procedure of leaf-mine histology”*).

### Phenological census of *B*. *euryae* larvae

To assess the number of generations per year and the duration of each stage of larval development, we carried out a phenology census of a wild population of *B*. *euryae*. Three *E*. *japonica* trees in the Kibogaoka Cultural Park were selected for the monitoring of phenological dynamics of *B*. *euryae* larvae in nature (S1 Fig). Trees less than two meters high were selected in order to allow us to inspect every leaf. The number of each instar larvae was recorded from June 2015 to October 2017 with approximately half-month intervals.

### Estimation of callus mass

To estimate the mass of callus present in inflated mines of fourth instar, 47 leaves mined by fourth instar larvae and 49 intact leaves with similar size were collected from two trees. The collected mined and intact leaves were weighed and measured their length and width. The length and width of each mine were also measured in order to estimate their surface areas. The leaf and mine shapes were respectively approximated by two circular segments and by an ellipse (S2 Fig). The of larvae were weighed at about 0.5 mg. Given that it represents less than 1% of plant tissues of the mine, the effect of larval presence on mined-leaf mass can be neglected. The leaf area density of mined and intact leaves was calculated by dividing their mass by their calculated surface. The average callus mass per mine was estimated by multiplying the difference between leaf area density of mined and intact leaves with the average mine surface.

### Exp. 1: Detached leaves experiment

To test whether *B*. *euryae* larval development requires plant resources that are not contained in the mined-leaves, contrary to classical leaf-miners, mined-leaves of every instar were detached and the development and the viability of larvae were recorded. Ten *E*. *japonica* trees were selected for this experiment (S1 Fig). Leaves mined by first, second, third or fourth instar larvae were detached from the ten trees. Five mined leaves per tree were collected for each instar, and each detached leaf hosted only a single larva (n_instar_ = 50, S3 Fig). Detached leaves were immediately transferred to the laboratory. Mined leaves were maintained in a centrifuge tube (118-mm long, 28-mm diameter) and a 2% sucrose solution was added to the leaf stalk that were wrapped with wiping paper to keep the leaves fresh [17]. Each larva was reared and the stage of death or adult emergence was recorded. Larvae that were parasitized by natural enemies (wasps) were removed from the data. All rearings and subsequent experiments were conducted at 25±1 ˚C under photoperiod of 16-h light: 8-h dark. This experiment was conducted from August to September in 2015 and from July to October in 2016.

To verify whether detached mined-leaves at third instar affect callus development that normally occurs after molting into fourth instar, histological cross-sections of fourth instar mines that had been detached at the third instar were made (n = 3) (for description of the method, see section below “*General procedure of leaf-mine histology”* first paragraph).

### Exp. 2: Larval translocation experiment

We aimed to test whether the absence of callus development in leaf-mines detached before the larvae reach the fourth instar (see Results) is sufficient to explain the larval mortality in leaf-mines (see Results). For this purpose, we conducted a translocation experiment. The experiment consisted of letting larvae in mined leaves detached at the third instar, grow until reaching the fourth instar, and of transferring them into vacant fourth instar mines freshly detached (S3 Fig). Third instar mines collected from the wild were reared in the laboratory as the detached leaf experiment, and then each reared mine was opened when the larva reached the fourth instar and carefully extracted the larva with a small brush to translocated into a vacant fourth instar mine. In order to prepare the vacant destination mines, mature fourth instar mines were collected from the wild at each translocating day. The fourth instar mines were made vacant by making a small aperture in the mine with a surgical scalpel and removing the fourth instar larva with forceps just before the translocation (S3 Fig). The only difference from the leaf-mines detached before inside larvae molted into the fourth instar is the presence of callus in the mine in which the larva was transferred, thus a recovery of a normal viability into adult would mean that callus is necessary to fourth instar development. As a control, to evaluate the effect of transfer on larval viability, fourth instars directly collected in the study site were translocated into fresh vacant fourth instar mines. The viability until adulthood was recorded in both conditions and was compared with of the viability in the detached leaves experiment (Exp. 1). This experiment was conducted from June to October 2016. Mines containing translocated larvae were reared in the laboratory as the detached leaf experiment.

### Exp. 3: Larval ablation experiment

To test whether the fourth instar larva directly induces callus development, late third instars were killed before they molt in to the next stage and the mine-structure was observed fourteen days later. Because an inflation is normally noticed after this time, an absence of callus formation would be interpreted as evidence of a direct role of the fourth instar larva in the induction of tissue proliferation. Larvae were killed with a thin needle. Histological cross-sections were performed on mines of late third instars, fourteen days after killing larvae (n = 3). As a control, cross-sections of fourth instar mines fourteen days after they molted from third instar (n = 3) were performed (for description of the method, see section below “*General procedure of leaf-mine histology”* first paragraph).

In order to test whether the induction of callus development is the result of a short event at the beginning of the fourth instar or a continuous process during this developmental stage, we tested if callus amounts are correlated with the time elapsed after beginning of fourth instar or with the duration of the interaction with the fourth instar larva. For this purpose, larvae inside mines were killed at three, six and ten days after molting into the fourth instar and, in each case, a histological cross-section was made fourteen days after molting into fourth instar (for description of the method, see section below “*General procedure of leaf-mine histology”* second paragraph). If callus formation is caused by a single early event, the amount of callus is expected to be the same in each condition, if depletion due to larval feeding is negligible, or to decrease as the duration of fourth instar increases, if insect feeding has a significant effect on the amount of callus. On the contrary, a positive correlation with the duration of fourth instar would be interpreted as evidence of a continuous induction process through the fourth instar larval life.

Larvae were killed by stabbing with a tiny needle from outside mines. As a negative control larvae were killed at the end of third instar and left mines for fourteen days on host plants before fixation. Callus quantity was estimated by dividing the area of callus on light micrographs by the mine width. This experiment was conducted in autumn 2017.

### General procedure of leaf-mine histology

Cross sections of about 2 mm width and 4 mm length were cut and were fixed for 4 h with 2.5% paraformaldehyde and 0.4% glutaraldehyde in 0.1 M McIlvaine citrate-phosphate buffer, pH 7.0. After dehydration in a graded series of ethanol, samples were embedded in medium grade LR White resin (London Resin Company Ltd, UK). Semi-thin sections (1 μm thick) were collected with a diamond knife (Diatome, Biel, Switzerland) installed on an ultracut R microtome (Leica, Rueil-Malmaison, France), placed on slides, fixed by heating at 120°C for 2 min and stained with Toluidine Blue O 0,1% (w/v in water) (Sigma-Aldrich, T0394).

Cross sections for the ablation experiment were cut and fixed with the same protocol but were included in paraffin. Section of 10μm were made with a rotary microtome (369 Yamato Kohki, Japan) and stained with Toluidine Blue O 0,1% (w/v in water) (Sigma-Aldrich, T0394).

Images were assembled using MosaicJ plugin of ImageJ [18] and treated with Photoshop and Lightroom (Adobe) in order to adjust contrast and white balance, and for cropping.

### Statistical analyses

Leaf area densities were compared using Welch’s *t*-test. The variations of leaf mass as a function of leaf area in mined and unmined leaves were compared using an analysis of covariance (ANCOVA). Results of the detached experiment were analyzed with the generalized linear model (GLM) using binomial probability distributions. The effect of variable among the ten particular “trees” and of detaching “instars” was tested on a response variable “viability until adulthood”. The differences in percentages of survival ratio until adulthood in the translocation experiments were analyzed using Fisher’s exact test, and the sequential Bonferroni correction [19] was applied to the *P*-values to keep the significance level at 0.05 throughout the multiple comparisons. The corrected *P*-values were indicated as *P* (*adjusted*). For the larval ablation experiment, a Spearman’s rank correlation coefficient between the increase of callus area and the duration of fourth instar was calculated. All statistical tests were carried out using the R package version 3.2.3 (R Developmental Core Team, 2016).

## Results

### *B*. *euryae* larval development

**Fig 1:**
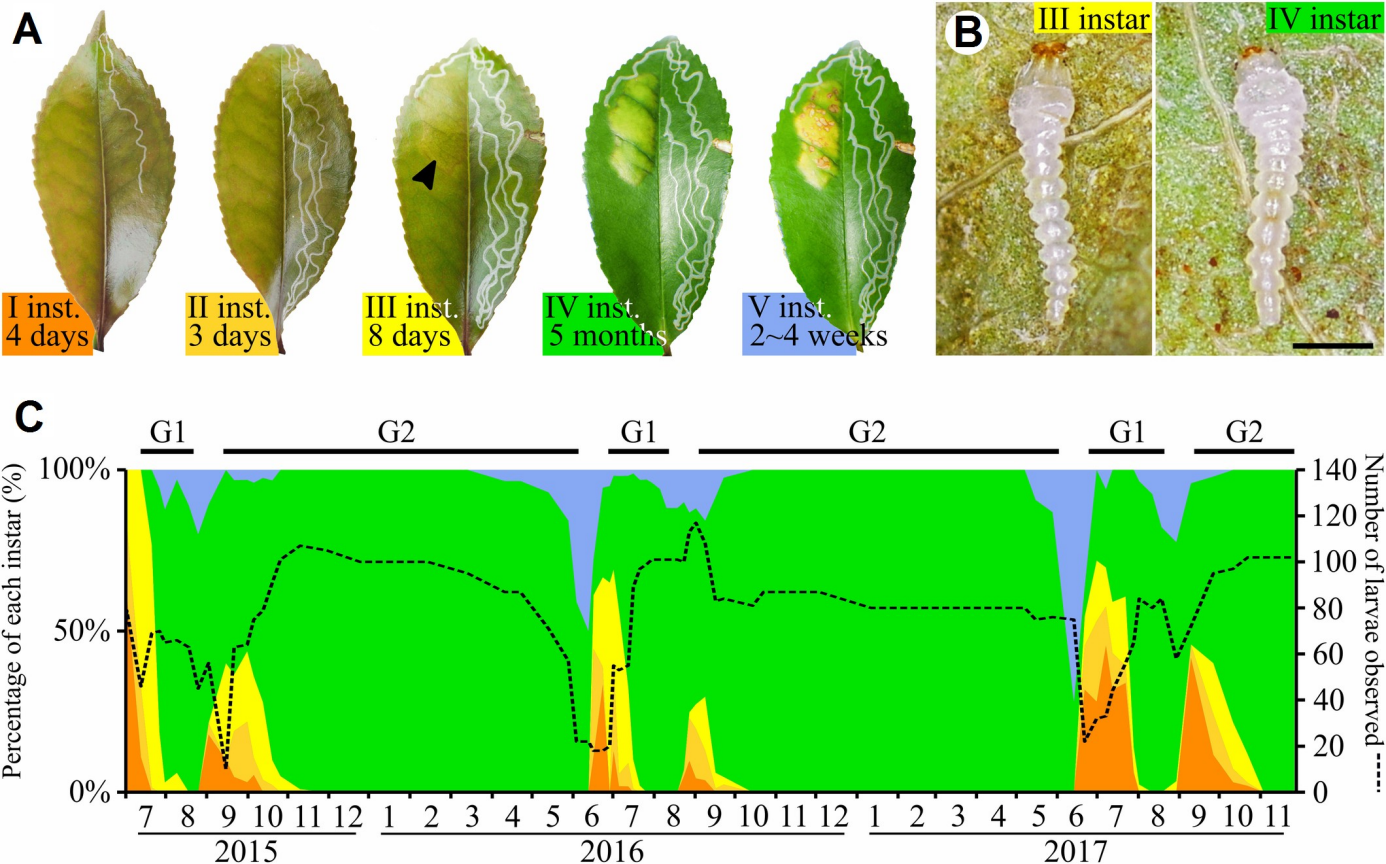
Life history and phenology of *Borboryctis euryae*. (A) The leaf-mines made by the five larval instars of *B*. *euryae* on *Eurya japonica*. First and second instars, respectively in dark and light orange and lasted 4 and 3 days, make a serpentine mine in the adaxial epidermis. The third instar (yellow) burrow a blotch mine (black arrow), visible by a brightening of leaf color and lasted 8 days. The fourth instar (green) is associated with an inflation and a progressive yellowing of the blotch mine. It lasted about five months in our rearing. The last stage (blue) lasted from 2 to 4 weeks and was linked with the apprearance of brown patches on the mine. (B) The morphology of third and fourth instar larvae. The mandibula shape, visible as the red structure on larva head, designate by its elongation a fluid-feeding habit for the third instar whereas its compactness for the fourth instar correspond to a tissue-feeder mode. Scale: 500μm. (C) B. euryae phenology. The left axis show the percentage of each instar recorded at each time point (same color code as in (A). The right axis corresponds to number of larvae at each count (dashed line). Instar abundances show the occurrence of two generation each year (G1 and G2) and the fact that fourth instar percentage do not reach zero at the end of G1 indicate that a part of the population is univoltine.

Eggs hatched three days after oviposition. Development time for first, second and third instars were four, three and eight days respectively (Fig 1A). Whereas first and second instars made a serpentine mine, the third instar expanded its gallery into a blotch mine. Mine inflation appeared at the fourth instar and concurrently with the hypermetamorphosis that involved large morphological changes with a shift from fluid-feeder to tissue-feeder mouthparts (Fig 1B) [20]. The blotch mine gradually inflated and turned to pale yellowish color about eleven days later. The fourth instar contrasted with other larval instar by its duration. During our greenhouse rearing, it lasted about five months before molting into fifth instar. This last fifth developmental stage took between two and four weeks (the imprecision was due to the difficulty to assess the instar transition due to the thickness of the mine tissues). It wass marked by the appearance of brown patches on the adaxial surface of the mine. Then the larva made a hole to exit the mine and spined a cocoon.

### Phenology census

Field observations revealed two occurrences of first-instar larvae each year, from mid-June to July and from mid-August to September, indicating that *B*. *euryae* has two generations per year (Fig 1C). However, about half of fourth instars did not molt into fifth instar during summer 2016 and 2017, suggesting half of the population is univoltine. All larvae reached fourth instar before November and overwintered at this stage. The size of insect population remained stable during the three years of recording, with about 100 individuals during winter period.

### Histological study of the early steps of the mine inflation

**Fig 2:**
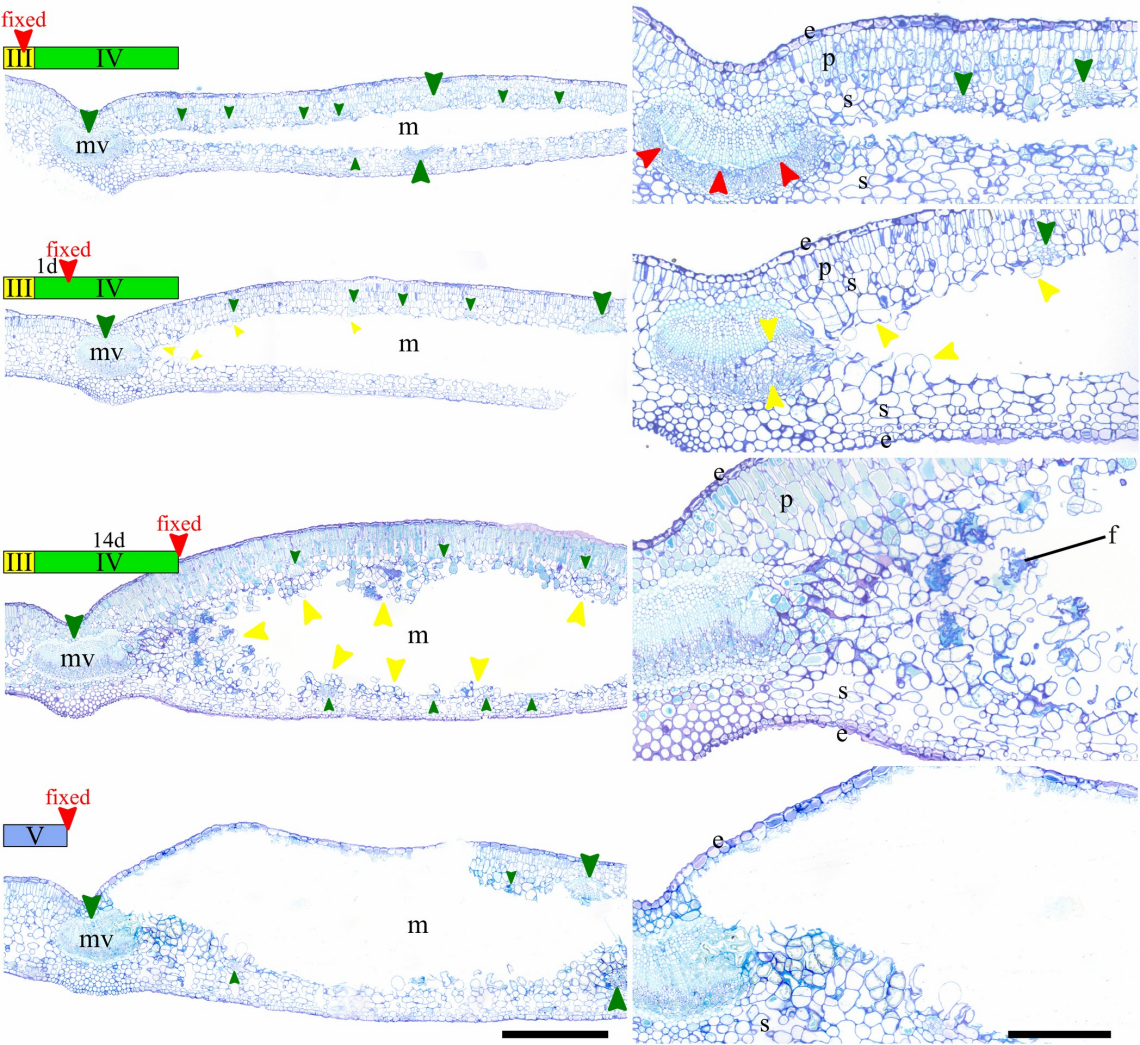
Histology of *Borboryctis euryae* leaf-mines on *Eurya japonica* in normal conditions. Cross-sections of mined leaves (left) and detail (right). From top to bottom, fixed at third instar, at one day after beginning of fourth instar, t fourteen days after beginning of fourth instar, at fifth instar. Legend: e: epidermis, p: palisade parenchyma, s: spongy parenchyma, f: larva frass, mv: mid-vein, m: mine, large green arrow: primary and secondary vein, small green arrow: other vein, yellow arrow: callus, red arrow: cut inside the mid-vein. Scale: 500μm (left), 200μm (right). Staining: Toluidine Blue O.

Third instar mine sections showed that the gallery is cut longitudinally through the superior half of the spongy parenchyma (Fig 2). As a consequence, large veins were sectioned in their middle and smaller ones were cut in their inferior part. A cut was done inside the mid-vein along the cambial layer. The parenchyma did not show any trace of feeding, which was consistent with the fluid-feeder mouthparts observed on third instar larvae. In the young fourth instar mine, the cut inside the primary vein was filled by hypertrophied cambial cells in division towards the mine gallery. Several cells showed mitotic activity close to the vascular system. At fourteen days after beginning of the fourth instar, an intense mitotic activity was present close to the mid-vein, and secondarily at the vicinity of each vascular element. As this growing mass parenchyma cells showed no apparent organization, this tissue can be considered as a callus. The presence of frass containing undigested cell wall confirmed the fourth instar is a tissue feeder. The absence of parenchyma destruction and the presence in the callus of broken cells indicated that the fourth instar exclusively feeds on the callus at this stage. On the contrary, the cross-section of fifth instar mine revealed patches of destruction of adaxial parenchyma, including palisade parenchyma, which showed that this developmental stage was feeding on those tissues. Those “feeding windows” corresponded with the brown patches noticed on the adaxial side of the mine (Fig 1A).

### Estimation of callus mass

**Fig 3:**
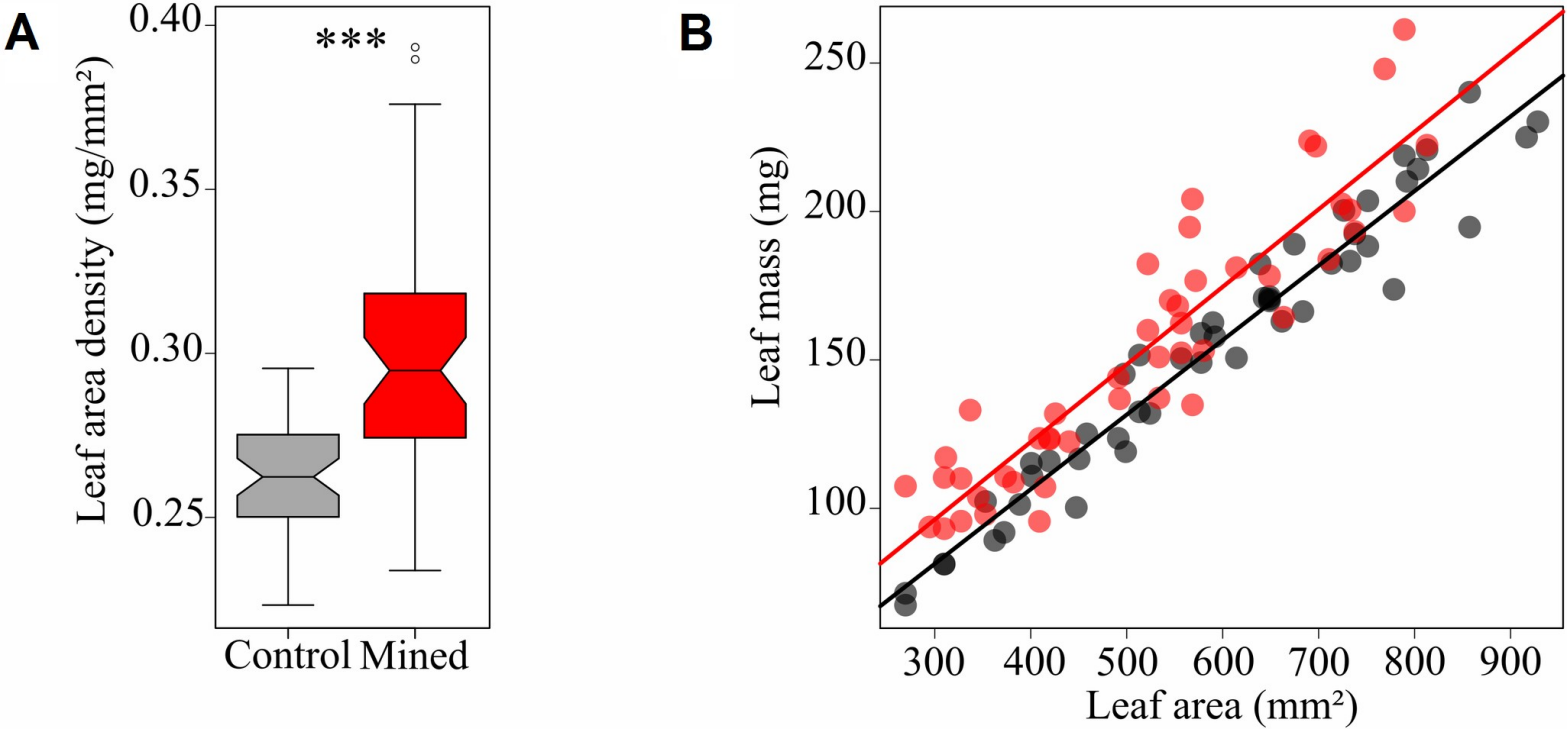
Estimation of the mass of callus in *Borboryctis euryae* leaf-mines on *Eurya japonica*. (A) Comparison of leaf-area density between leaves with fourth instar mines (red) and intact leaves (black), and (B) evolution of their mass in function of leaf area.

The area density of mined leaves was superior to the unmined (*t* = -6.2166, df = 62.656, *p*-value = 4.612e-08) (Fig 3A). The coefficients between leaf area and leaf mass were similar between mined and unmined leaves, and there was no significant interaction between leaf area and the two conditions (mined or unmined) (ANCOVA, for leaf area × condition, *P* = 0.577) (Fig 3B). This suggests that the callus mass per mine is constant and is not a function of leaf area. The difference of intercept provided an estimation of the callus mass: 12.7mg per mine. After dividing by the average mine surface the callus amounts in the mine was estimated to represent an area density of 0.11mg/mm^2^, which corresponded to an increase of 45% compared to control leaf area density.

### Exp. 1: Detached leaves experiment

**Fig 4:**
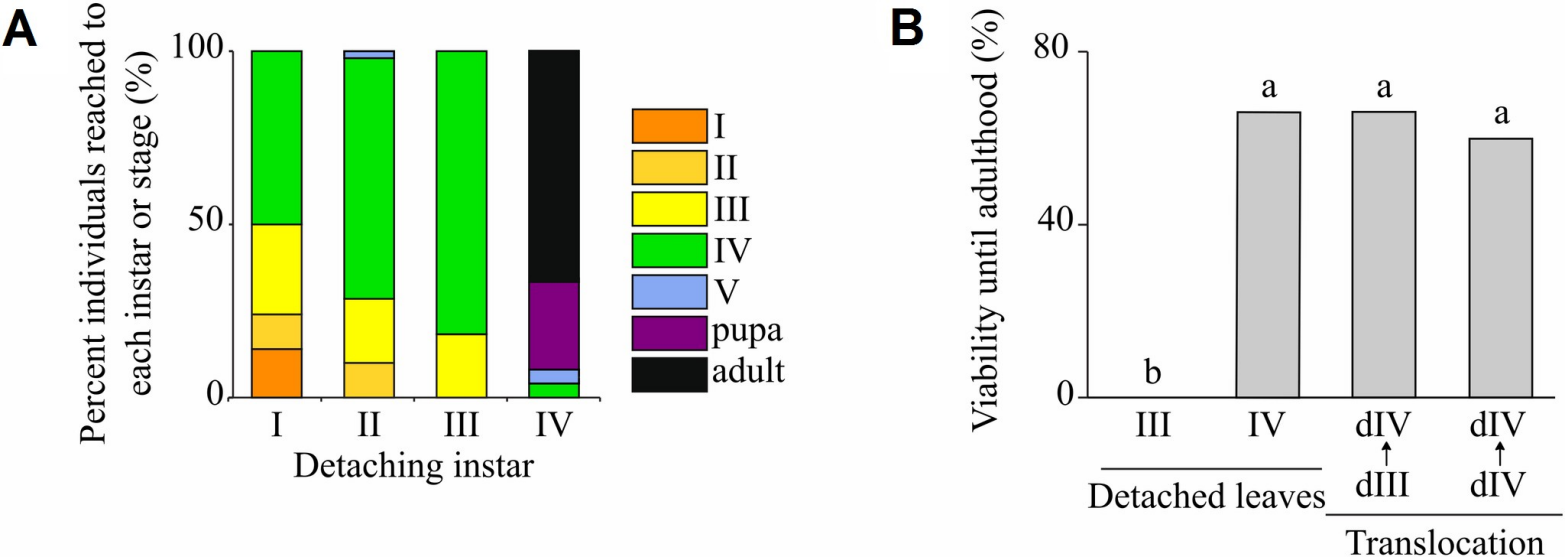
Effect of detaching *Eurya japonica* leaves mined by *Borboryctis euryae* on larval viability and callus development. (A) Last developmental stage reached after detaching a mined leaf, as a function of larval instar at leaf detachment, in percentage (Exp. 1: detached leaves experiment). (B) Viability until adulthood when mined leaf is detached at third and at fourth instar, and when larva is transferred at third and at fourth instar into a fourth instar mine, in percentage (Exp. 2: translocation experiment).

No larvae succeeded to develop into pupae when mined leaves were detached at first, second and third instars, and we did not notice any inflation of the detached leaves even when the inside larvae developed into fourth instar (Fig 4A). All larvae died before fifth instar, except one in a mined leaf detached at second instar that died just after molting into fifth instar. In contrast, in the case of leaves detached at fourth instar, 92% of the larvae successfully pupated and 66% could survive until adulthood. Significant effect of the detached instars was detected on the reached instars or stages (GLM, X^2^ = 63.312, *P* = 1.764e-15) and on the viability until adulthood (GLM, X^2^ = 105.439, *P* < 2.0e-16). There was no influence of the ten fixed trees on the reached instars or stages (GLM, X^2^ = 0.065, *P* = 0.7994) and on the viability until adulthood (GLM, X^2^ = 0.064, *P* = 0.7998). All detached leaves remained green until the end of insect life and did not present any sign of decaying.

### Exp. 1: Histology of detached third instar leaf-mine

**Fig 5:**
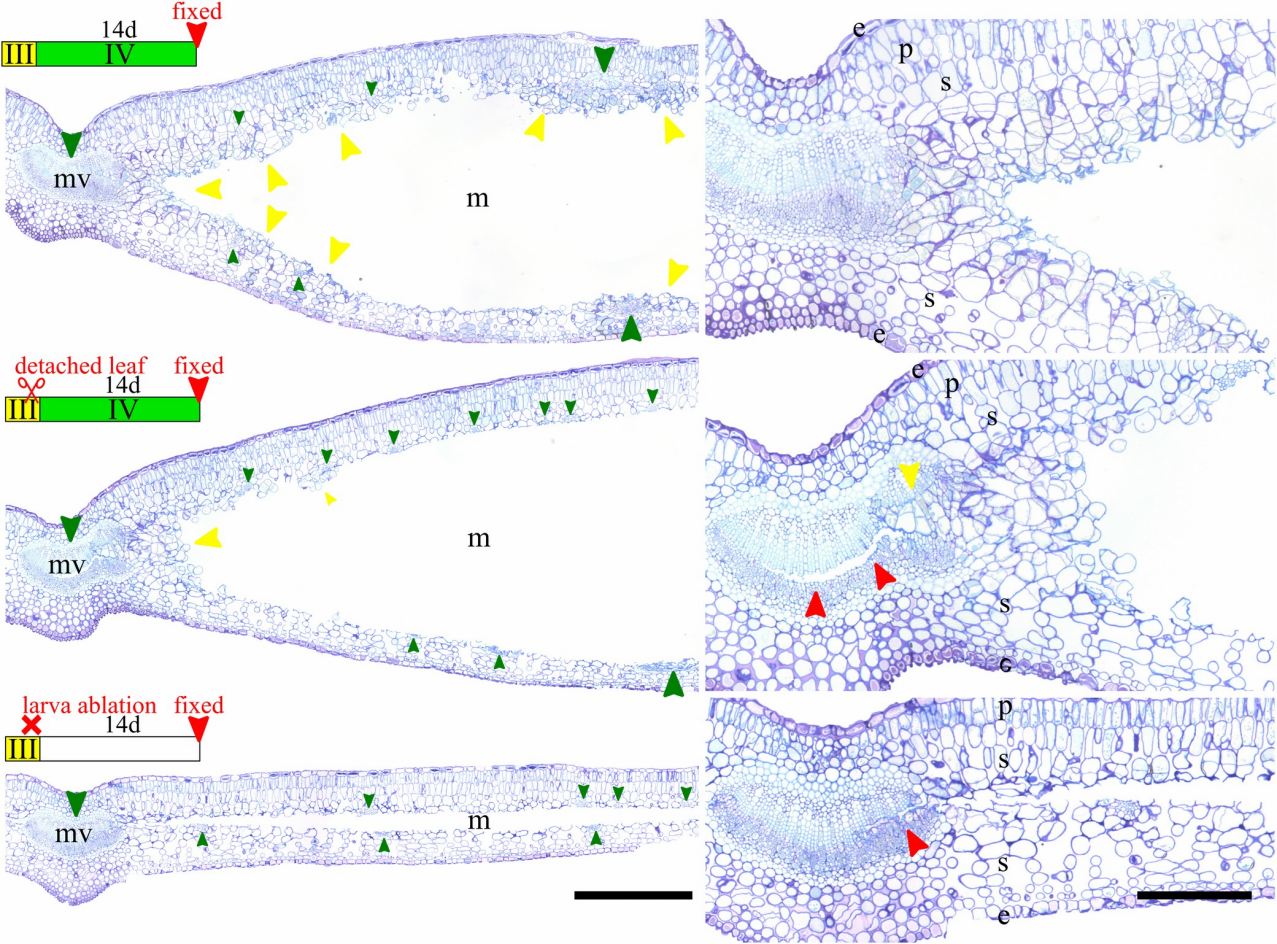
Histology of *Borboryctis euryae* leaf-mine on *Eurya japonica* after detaching the leaf and killing the larva. Cross-sections of mined leaves (left) and detail (right). From top to bottom, Fourth instar mine fixed at fourteen days after beginning of fourth instar (control), at fourteen days after beginning of fourth instar and after detaching the mined leaf at third instar (Exp. 1: detached leaves experiment), at fourteen days after killing the larvae at late third instar (Exp. 3: larval ablation experiment). Legend: e: epidermis, p: palisade parenchyma, s: spongy parenchyma, mv: mid-vein, m: mine, large green arrow: primary and secondary vein, small green arrow: other vein, yellow arrow: callus, red arrow: cut inside the mid-vein. Scale: 500μm (left), 200μm (right). Staining: Toluidine Blue O.

Callus proliferation was present in the leaf-mine, especially around the midvein (Fig 5). However, its abundance was considerably reduced compared to the control. In addition, no callus was observed around other vascular elements except in the vicinity of the midvein, and it filled only partially the midvein cut.

### Exp. 2: Larval translocation experiment

Contrary to those that were not translocated, where no one survived until pupation, 66% of larvae translocated into mines containing callus became adult, which represents statistically significant increase of viability (Fisher’s exact tests, *P* = 6.683e-14 < *P (adjusted)*) (Fig 4B). Their viability was neither statistically different from those translocated between two mines containing callus (Fisher’s exact tests, *P =* 0.679 > *P (adjusted)*), nor from those reared from mined leaves detached at fourth instar (Fisher’s exact tests, *P =* 1 > *P (adjusted)*).

### Exp. 3: Histology of leaf-mines after larval ablation

**Fig 6:**
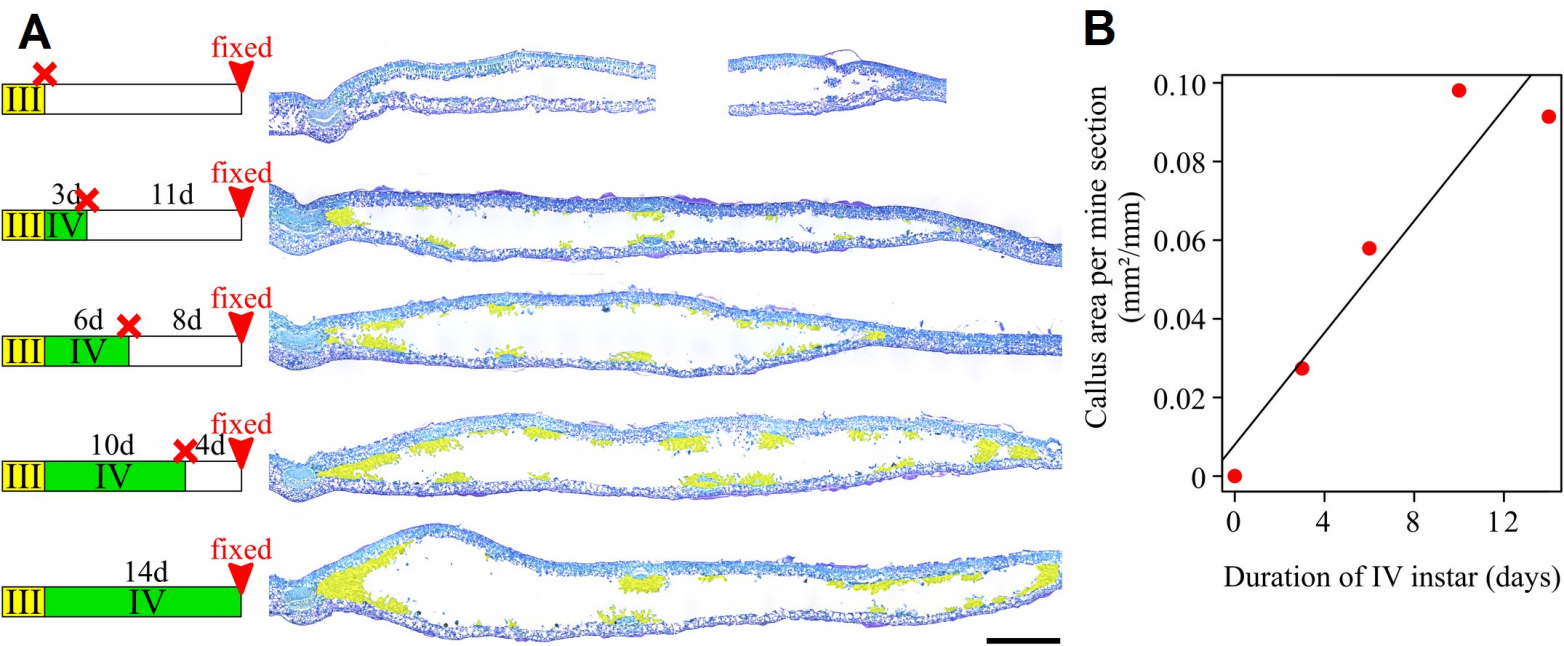
Increase of callus tissue in the mine as a function of the duration of the interaction with fourth instar larva. (A) Cross-sections of mined leaves at fourteen days after beginning of fourth instar and when larva is killed three, six or ten days after beginning of fourth instar. Callus is highlighted in yellow. Scale: 1mm. Staining: Toluidine Blue O. (B) Callus area per mine section measured on histology sections.

Sections revealed an absence of callus formation when larvae were killed at third instar (Fig 5). The leaf-mine’s histology was similar in every aspect with that of third instar mines (Fig 2). In contrast, the callus increased for the section where larvae were killed after molting into fourth instar. Callus was mainly observed around main veins and increased until the ten days treatment in which high volumes of callus surrounded every vascular element (Fig 6A). Although the present small dataset did not allow us to conclude about the dynamics of callus formation, the correlation between the time spent by the fourth instar in the mine and the amount of callus in it was significant (r^2^ = 0.9630609, *P* = 0.008475) (Fig 6B).

## Discussion

Our results provide new insight into the function and the origin of gall-like structures observed in some leaf-mines. The presence of cell proliferation in some leaf-mines used to be interpreted as a wound reaction, probably stimulated by moisture condition in the mine [14]. Here we present the first case for which we can exclude physical conditions in the mine and tissue regeneration as the direct causes of callus formation, given that no callus develops in the absence of the fourth instar larvae. Furthermore, the newly formed tissues are usually considered as byproducts of leaf-miner feeding that do not count in their nutrient supply [14]. On the contrary, our results show that callus represents the unique food source of the fourth instar larvae and, as a consequence, is essential for *B*. *euryae* larval development. Finally, a direct role of the insect in the induction of this proliferative tissue, although suspected [14,15], has never been demonstrated before this study.

Therefore, the characteristics of *B*. *euryae* life-habit place it in the so-called “no man’s land” between leaf-miners and gall inducers [14]. Thus, it supports that leaf-miners and gall-inducers are part of a continuum of plant manipulation [21]. We propose to name this intermediate feeding strategy “mine-galling”, a term coined by [22]. Mine-gallers are defined as an endophagous herbivore that start and ends their interaction with their host plant by mining tissues, and induce the formation of a callus that is necessary for its subsistence. They differ from strict gall-inducers by the absence of induction of a new plant organ formation, like neovascularization and tissue differentiation. If those criteria are met, endophagous herbivores already mentioned as having callus in their galleries could be considered as mine-gallers. In particular, several other microlepidopteran may meet our definition [16,22,23]. We recently found other cases also belonging to the subfamily Acrocercopinae (Gracillariidae) as *Borboryctis* (unpublished data). We note that these insects have in common to make mines inside the mesophyll of sclerophyllous leaves or to bore through petioles, which suggests that callus development may require a confined environment with low evaporation, although those physical conditions are not directly involved in callus induction.

As mine-gallers share with the leaf-miners that induce a green-island phenotype the ability to enhance their nutritional environment [12], they may have some mechanisms of plant manipulation in common. It has been established that these insects maintain a photosynthetic activity in their mines in autumn by secreting cytokinins [24]. In addition, cytokinins and auxins are known to be produced by many gall inducers [25] and are essential for successful *in vitro* callus induction [26]. Future work should assess whether secreted phytohormones play a role in callus induction by *B*. *euryae*. The fact that callus development is not resulting from a single induction event but from continuous stimulation is compatible with regular phytohormone injection by the insect.

The nutritional quality of callus is also unclear. We show that it is essential for larval development from the fourth instar onwards. The fourth larval stage is the longest and more variable in duration, it feeds exclusively on callus and dies if callus is not produced. As a possible explanation, the callus may contain less toxic compounds like caffeine, known to be present in *E*. *japonica* leaves [27], than the mesophyll cells. Supplementary studies are needed to compare the metabolome and intracellular organelles of callus and those of the mesophyll.

Mine-gallers may constitute a transitional form in the evolution of gall-inducers from leaf-miners, an evolutionary path that is still poorly understood [28]. The lack of a robust phylogeny prevents us from inferring possible origins of this life-style in the case of *B*. *euryae*. However, our observations give elements to discuss its adaptive significance. Histological cross-sections of fourth instar mines reveal that callus formation at the edges of the mine, and particularly within the mid vein, cause the mine swelling (Fig 2). Now, creating a three-dimensional space in their mines is a common strategy in Gracillariidae, usually by making a tentiform mine [14], and could represent a way to avoid parasitoids (29). Given that the stiffness of *E*. *japonica* evergreen leaves could make it impossible to fold a tentiform mine like other Acrocercopinae, the mine-gall life-style may constitute an alternative to increase the mine volume in order to avoid or limit parasitoids attack, in addition to a nutritional enhancement. Indeed, of 200 larvae used in the detached experiment, only two larvae parasitised in the *B*. *euryae* population, and this parasitism rate is drastically lower than that of other Acrocercopine species [29].

Without excluding this adaptive hypothesis, the phenological census analysis allows us to formulate another one. Our results show that *B*. *euryae* overwinter as fourth instar inside the mine, which consequently can last up to eight months. Moreover, we found that a substantial part of the population was not bivoltine but univoltine, which means that maximal fourth instar duration could reach up to eleven months. Similarly, having different voltinism in one population is also known in some gall-inducing species [30,31]. These long durations for a leaf-miner instar may be allowed by the continuous regeneration of the callus induced by the insect. Like a cornucopia, the potentially unending nourishment provided by the mine to the fourth instar may constitute a considerable adaptive value, allowing *B*. *euryae* to have flexible larval development time. Contrary to other gall adaptive hypotheses that suppose that gall adaptive value may come from the better living conditions they provide compared to non-galling systems, i.e. a better nutrition quality, a better protection against enemies, or against environmental conditions [32], the “cornucopia hypothesis” assumes that the maintenance of favorable living condition provides an adaptive value to this life-style. This value may consist in buffering the impact of the variation of environmental conditions [31] or mitigating parasitoid impact on populations [30].

As a broader perspective, this system is particularly suitable for comparative approaches. Contrary to most gall-inducing insects for which plant manipulation starts with their interaction with their host plant, galls appear at a specific time of *B*. *euryae* development in the leaf. This characteristic allows intra-species comparison of gene expressions, a powerful method that could help to find factors of gall-induction.

## Supporting information

S1 Fig Location of the study site. Orange circles show trees used in phenology census and purple circles show trees used for detached leaf and larval translocation experiments.
(TIF)

S2 Fig Geometrical estimate of leaf and leaf-mine area. (TIF)

S3 Fig Plan of detached leaves and larval translocation experiments (Exp. 1 and Exp. 2). (TIF)

S4 Fig Replicates of the leaf-mines cross-sections of the detached leaf experiment (Exp. 1) and the larva ablation experiment (Exp. 3). Cross-sections of leaf-mines at fourteen days after beginning of fourth instar (Ctrl.), at fourteen days after beginning of fourth instar on a detached leaf (D.), and at fourteen days after killing the larvae at late third instar (Killed). Each mine has been cut close to midvein (C) at the mine edge (E). Scale: 500μm. Staining: Toluidine Blue O.
(TIF)

S1 Table The meteological data of the study site in 2015 to 2017. All data derived from the observation point of Japan Meteorological Agency in Higashiomi town, which is the nearest observation point to the study site.
(XLSX)
