# Supplementary figures and images for: Inside the horn of plenty: Leaf-mining micromoth manipulates its host plant to obtain unending food provisioning
Source: PLoS One. 2018 Dec 21;13(12):e0209485. doi: 10.1371/journal.pone.0209485 (PMC6303051; doi:10.1371/journal.pone.0209485)

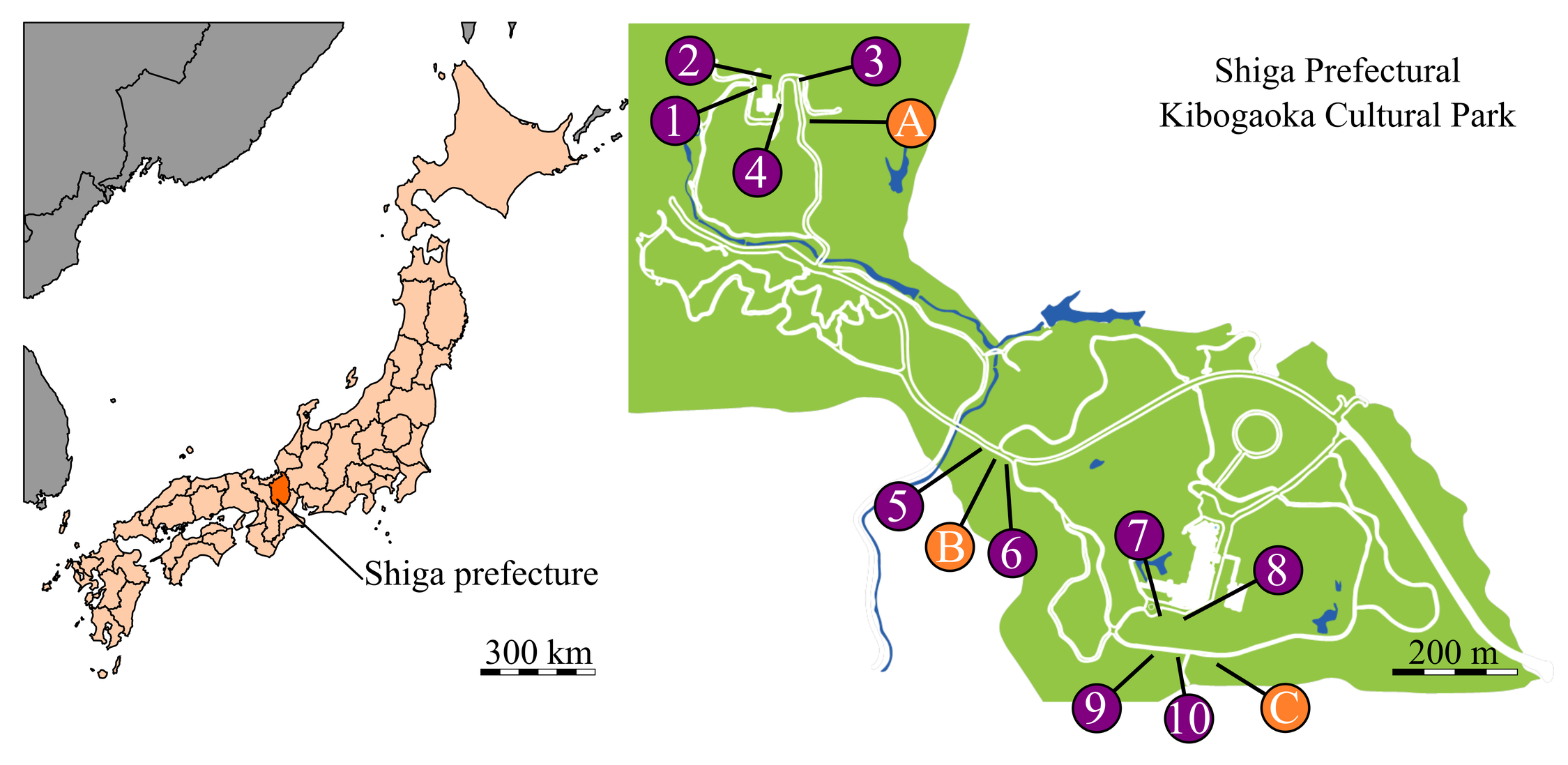

Supplement: S1 Fig — Orange circles show trees used in phenology census and purple circles show trees used for detached leaf and larval translocation experiments. (TIF) [file pone.0209485.s001.tif]

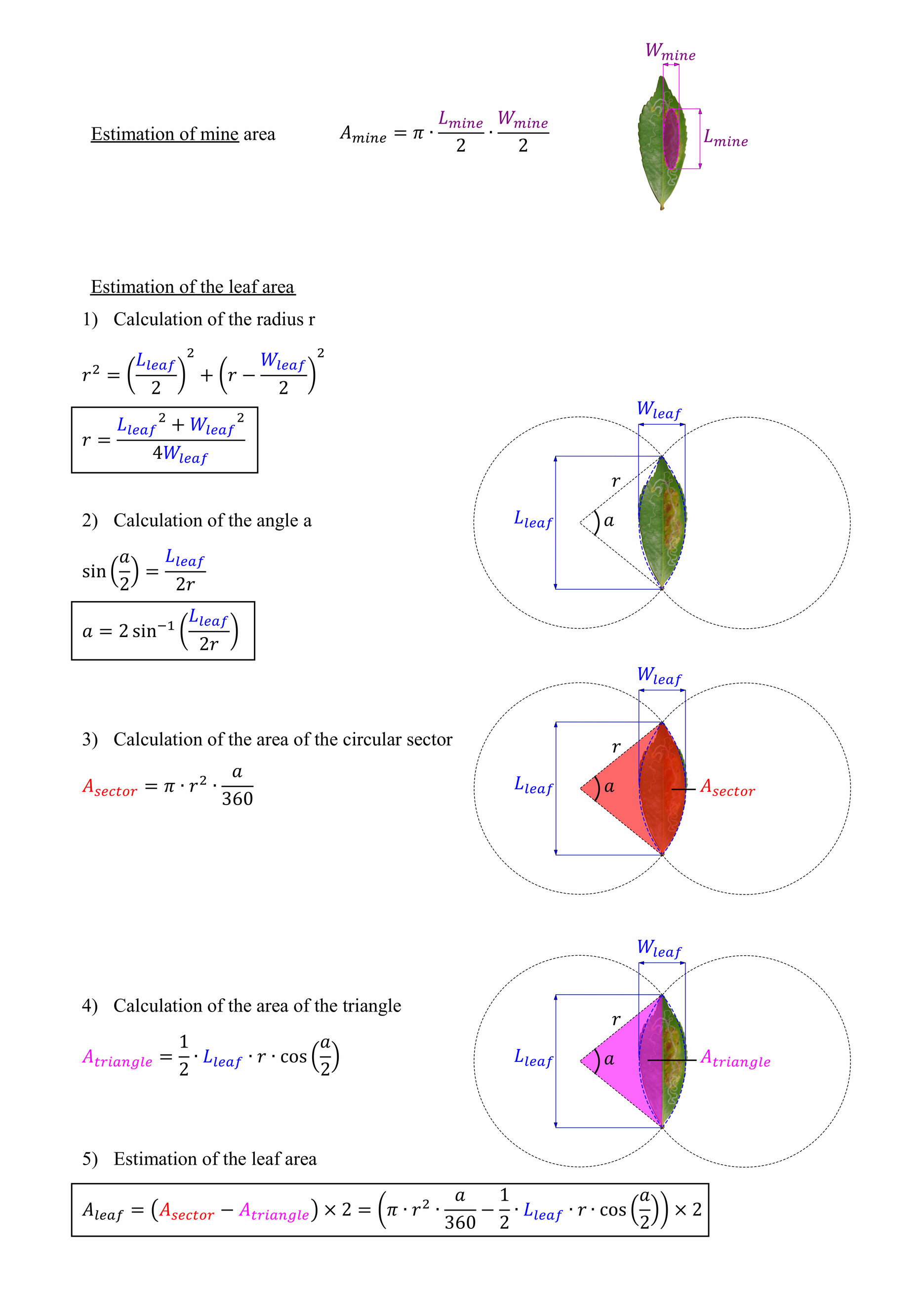

Supplement: S2 Fig — (TIF) [file pone.0209485.s002.tif]

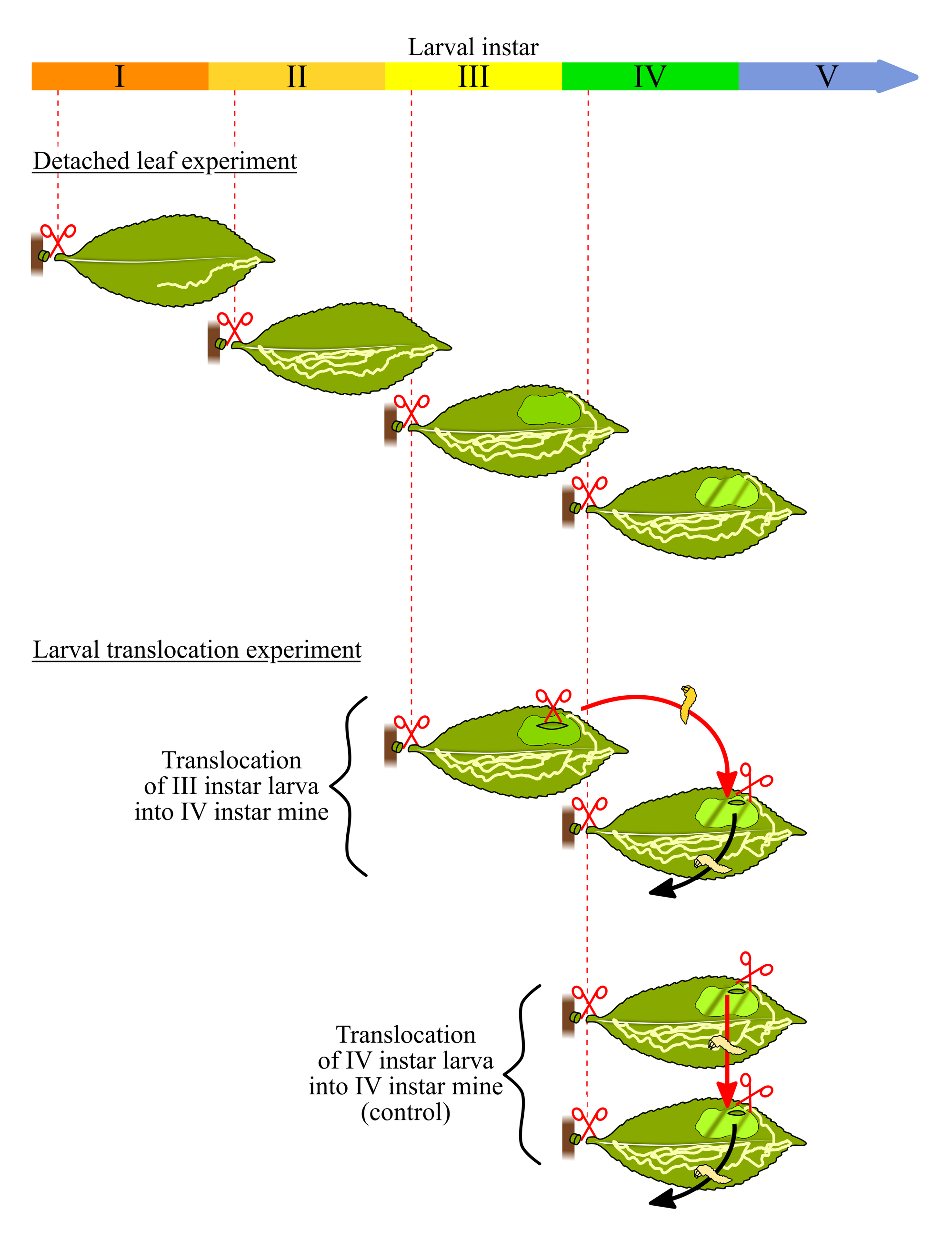

Supplement: S3 Fig — (TIF) [file pone.0209485.s003.tif]

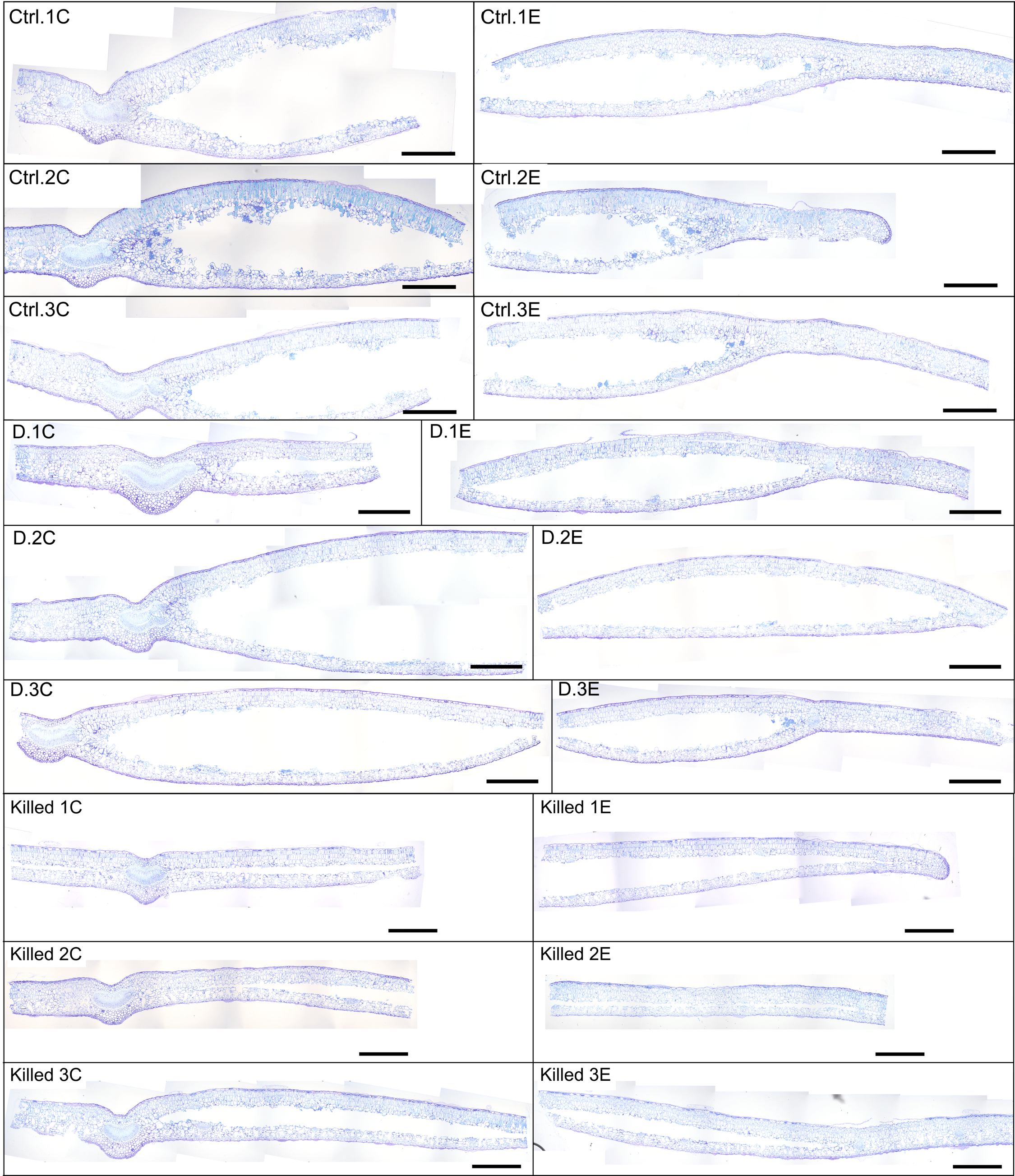

Supplement: S4 Fig — Cross-sections of leaf-mines at fourteen days after beginning of fourth instar (Ctrl.), at fourteen days after beginning of fourth instar on a detached leaf (D.), and at fourteen days after killing the larvae at late third instar (Killed). Each mine has been cut close to midvein (C) at the mine edge (E). Scale: 500μm. Staining: Toluidine Blue O. (TIF) [file pone.0209485.s004.tif]
